# The Protection of *Lactiplantibacillus plantarum* CCFM8661 Against Benzopyrene-Induced Toxicity *via* Regulation of the Gut Microbiota

**DOI:** 10.3389/fimmu.2021.736129

**Published:** 2021-08-10

**Authors:** Leilei Yu, Lingyu Zhang, Hui Duan, Ruohan Zhao, Yue Xiao, Min Guo, Jianxin Zhao, Hao Zhang, Wei Chen, Fengwei Tian

**Affiliations:** ^1^State Key Laboratory of Food Science and Technology, Jiangnan University, Wuxi, China; ^2^School of Food Science and Technology, Jiangnan University, Wuxi, China; ^3^National Engineering Research Center for Functional Food, Jiangnan University, Wuxi, China

**Keywords:** probiotic, *Lactiplantibacillus plantarum*, benzopyrene, gut microbiota, SCFAs, gut barrier, behavioral performance

## Abstract

The present study evaluated the protection of *Lactiplantibacillus plantarum* CCFM8661, a candidate probiotic with excellent benzopyrene (B[a]P)-binding capacity *in vitro*, against B[a]P-induced toxicity in the colon and brain of mice. Mice that received B[a]P alone served as the model group. Each mouse in the *L. plantarum* treatment groups were administered 2×10^9^ colony forming unit (CFU) of *L. plantarum* strains once daily, followed by an oral dose of B[a]P at 50 mg/kg body weight. Behavior, biochemical indicators in the colon and brain tissue, and the gut microbiota composition and short-chain fatty acid (SCFA) levels in the gut were investigated. Compared to the treatment in the model group, CCFM8661 treatment effectively reduced oxidative stress in the brain, improved behavioral performance, increased intestinal barrier integrity, and alleviated histopathological changes in mice. Moreover, CCFM8661 increased the gut microbiota diversity and abundance of *Ruminococcus* and *Lachnospiraceae* and reduced the abundance of pro-inflammatory *Turicibacter* spp. Additionally, the production of SCFAs was significantly increased by *L. plantarum* CCFM8661. Our results suggest that CCFM8661 is effective against acute B[a]P-induced toxicity in mice and that it can be considered as an effective and easy dietary intervention against B[a]P toxicity.

## Introduction

Benzopyrene (B[α]P) is a kind of polycyclic aromatic hydrocarbon that is categorized as a group I carcinogen by the International Agency for Research on Cancer (1, 2). Contaminated food is an important source of human exposure to B[α]P, which is up to 125 ng per day for each person (2, 3). B[α]P would be produced during food processing, such as smoking, grilling, frying. B[α]P-contaminated foods include vegetables (13 ng/kg), cereals (262 ng/kg), smoked fish (800-13900 ng/kg), and dairy products (11-78 ng/kg) (4). After entering into the host’s body, B[α]P would bind to the aryl-hydrocarbon receptor and be activated by cytochrome P-4501A1 (CYP1A1) to produce immense size DNA adducts and ROS (2, 5), which would inactivate tumor suppressor genes or activate oncogenes, resulting in DNA damage, mutation, and cancer (6, 7).

B[α]P is neurotoxic and it affects the levels of 5-hydroxytryptamine, 5-hydroxyindoleacetic acid, and target protein kinase C (8), leading to neurotoxicity and behavioral disturbances (6, 9). More seriously, the neurotoxicity of B[α]P has a genetic effect (10). However, the effects of B[α]P on the gut have not been thoroughly studied so far. The intestinal tract is the first vital barrier against oral B[α]P exposure. Therefore, it is necessary to investigate the negative effects of B[α]P on the gut, especially the gut microbiota, gut barrier and intestinal histopathology. Previous researches have reported the harmful effects of B[α]P on the intestinal barrier, such as changes in the expression levels of tight junction (TJ) proteins (11, 12). Ribiere et al. demonstrated that B[α]P exposure dramatically changed the gut microbiota composition, thereby causing a pro-inflammatory intestinal environment and leading to moderate inflammation in ileum and colon of mice (13). Moreover, the important roles of intestine microbiota and SCFAs in regulating host health have aroused the increasing concern (14–16). For example, gut microbiota-generated SCFAs promote metabolism *via* gut-brain axis, thereby reducing body weight, adiposity, and blood glucose (15).

Lactic acid bacteria (LAB), a group of safe gram-positive microorganisms, are widely used in various fermented food (17). LAB are also the important components of the human gut microbiota with various physiological function, including balance of immune cell, regulation of metabolites and gut microbiota. *Lactiplantibacillus plantarum* is a super vital LAB that is capable of binding or sequestering various carcinogens *in vitro* and *in vivo* and is a safe and cost-effective intervention way (18–20). Apart from its capacity to bind carcinogens, *L. plantarum* regulates the gut microbiota and has antioxidative properties, which may be important for the alleviation of B[α]P toxicity. Therefore, the aim of this study was to select a novel probiotic strain with excellent B[α]P-binding ability from the 23 *L. plantarum* strains tested and evaluate its protective effects on the colons and brains of mice exposed to B[α]P, especially the impacts on the intestinal microbiota composition and intestinal barrier function. Moreover, possible protective mechanisms against B[α]P toxicity are proposed.

## Material and Methods

### Bacterial Strains and Culture

Twenty-three *Lactiplantibacillus plantarum* strains, including *L. plantarum* CCFM571, CCFM595, CCFM8610, CCFM438, CCFM726, CCFM408, CCFM8661, CCFM634, CCFM175, CCFM242, CCFM361, CCFM259, CCFM382, Lp45, FJSWX14-5, DYNDL58M4, PS3-9, FFJND7-L5, HY9-10, M2-05-R02, FJSZJ4-L5, 4L-4 and VNMWLT1M12, were obtained from Research Center of Food Biotechnology in Jiangnan University (Wuxi, China). The cultivation of all *L. plantarum* strains was in MRS broth (Hopebio, Qingdao, China) at 37°C for 18h.

### Determination of the B[α]P-Binding Capacity of *L. plantarum* Strains *In Vitro*

The B[α]P-binding ability of the 23 *L. plantarum* strains was estimated as previously described (21). The cultured biomass was centrifuged at 5,000 × *g* for 15 min and washed twice with ultrapure water to acquire cell pellets. The cell pellets were re-suspended in the ultrapure water containing 10µg/mL B[α]P (Sigma-Aldrich, St Louis, MO, USA). The suspension was centrifuged after incubation for 2h at 150 rpm and 37°C, and the residual B[α]P concentration in the supernatant was analyzed. B[α]P levels were measured using a high performance liquid chromatography (HPLC) equipped with a Waters Atlantis C18 reverse-phase column (4.6×250 mm×5 µm, 30°C; Waters Corporation, Milford, MA, USA). The mobile phase was acetonitrile:water (88:12). The injection volume was 20μL, with a flow rate of 1mL/min, and the fluorescence detection wavelength was 406 nm.

The B[α]P-binding abilities of the *Lactiplantibacillus* strains are expressed as the B[α]P removal rate, which was calculated as follows:

Removal rate (%)=[(C_i_–C_r_)/C_i_]×100%, C_i_ and C_r_ are the initial and residual B[α]P level, respectively.

### Animal Experimental Procedure

Male adult BALB/c mice (8-week-old) were purchased from Slack limited company (Shanghai, China). Mice were kept in cages at a constant temperature (22°C ± 1°C) and humidity (55% ± 10%) under a 12-h/12-h light/dark cycle and had free access to food and water. All procedures and protocols of mice experiments were performed according to the guidelines of the Animal Care and Use Committee and the Ethics Committee of Jiangnan University (JN.No20190915b0481210).

The mice were divided into four groups and allowed to acclimatize to their environment for 1 week. The experimental schedule is shown in **Table 1**. Group 1 (control group) was administered skim milk and corn oil without B[α]P. Group 2 (model group) was administered B[α]P dissolved in corn oil at a dose of 50 mg/kg b.w. Groups 3 and 4 (CCFM8661 and CCFM382 groups, respectively) were administered 2×10^9^ CFU of *L. plantarum* strains CCFM8661 and CCFM382, respectively, and 50 mg/kg b.w. of B[α]P. All treatments were administered *via* oral gavage for 5 weeks. Mice were fasted for 12h before sacrifice. Blood samples were collected and centrifuged at 3,000 × *g* for 15 min to obtain the serum, which was used for biochemical analysis. Colon and brain tissues were immediately washed with 0.9% saline and separated into two parts: one part was stored at -80°C for subsequent measurements, and the other part was fixed with 4% formalin for histopathological analysis.

**Table 1:** Animal experimental protocol.

Group (n = 8)	Treatment (5 weeks)
Control	SM+CO
Model	SM+B[α]P
CCFM8661	*L. plantarum* CCFM8661+B[α]P
CCFM382	*L. plantarum* CCFM382+B[α]P

CO=0.2 mL corn oil; SM=0.2 mL skim milk; B[α]P=0.2 mL corn oil containing 50 mg/kg body weight of B[α]P; CCFM8661 = 0.2 mL skim milk containing 2×10^9^ CFU of L. plantarum CCFM8661; CCFM382 = 0.2 mL skim milk containing 2×10^9^ CFU of L. plantarum CCFM382. Animals received corn oil, skim milk, B[A]P, and L. plantarum strains via gavage.

### Open-Field Test

The test was performed as previously described with minor modifications (22, 23). The size of apparatus used for the open-field test was 50cm×50cm square with four white walls. The edge region is a 15cm area near the walls and the rest of the field was the central area. Each mouse can move freely within the apparatus for 15min. A camera was used to record their movements, and the data were analyzed using EthoVision (Noldus, Wageningen, Netherlands). The apparatus was cleaned with 75% ethanol after each test to eliminate any possible odor cues. The total distance traveled and the times spent in the center and the edge regions were calculated to measure anxiety-like behavior.

### Determination of 3-OH B[α]P Levels in Feces

The mouse feces were mixed with acetonitrile (1:2) by vortex oscillation. After centrifugation at 10000 rpm for 10 min, the 3-OH B[α]P levels in supernatant was analyzed by HPLC-fluorescence detection (24). The mobile phase was methanol:water (97:3, pH4.5). The injection volume was 20 μL, with a flow rate of 0.5 mL/min, and the fluorescence excitation and emission wavelength were 365 and 450 nm, respectively.

### Determination of Oxidative Stress-Related Parameters in the Brain

The MDA level and SOD activity were measured using ELISA kit according to the operating instructions of the manufacturer (Jiancheng Bioengineering, Nanjing, China).

### RT-qPCR Analysis

Colon and brain tissue (0.1g) samples were lysed in TRIzol reagent (Ambion, USA) for RNA extraction. cDNA was synthesized using the RevertAid (Thermo Fisher Scientific, Waltham, MA, USA). Gene expression levels were determined using validated primers for *Gapdh*, *Zo-1*, *Occludin*, *Claudin-1*, *CYP1A1*, *Bax*, *Bcl-2*, and *p53* (**Table 2**) and iTaq Univeral (Bio-Rad, Hercules, CA, USA) on an RT-qPCR system (BioRad-CFX384) (25). The PCR program comprised initial denaturation at 95°C for 2 min, followed by 40 cycles of 95°C for 30 s, 60°C for 30 s, and 72°C for 30 s, and finally, 72°C for 5 min. Relative quantification of these target gene expression levels was performed after normalization to *Gapdh* gene expression levels using the 2^−ΔΔCt^ method.

**Table 2:** Primer sequences used for RT-qPCR.

Primer	Sequence (5’-3’)	
GAPDH	Forward (F)	TGCACCACCAACTGCTTAG
	Reverse (R)	GATGCAGGGATGATGTTC
ZO-1	F	CTTCTCTTGCTGGCCCTAAAC
	R	TGGCTTCACTTGAGGTTTCTG
Occludin	F	CACACTTGCTTGGGACAGAG
	R	TAGCCATAGCCTCCATAGCC
Claudin-1	F	GATGTGGATGGCTGTCATTG
	R	CCTGGCCAAATTCATACCTG
CYP1A1	F	CCTCATGTACCTGGTAACCA
	R	AAGGATGAATGCCGGAAGGT
Bax	F	CTACAGGGTTTCATCCAG
	R	CCAGTTCATCTCCAATTCG
Bcl-2	F	GTGGATGACTGAGTACCT
	R	CCAGGAGAAATCAAACAGAG
P53	F	GTATTTCACCCTCAAGATCC
	R	TGGGCATCCTTTAACTCTA

### Histopathological Analysis

Colon tissue was fixed in 10% formalin saline for 24 h and then embedded in paraffin. The paraffin was sliced into 5-μm-thick sections. After sectioning, the tissue samples were stained with H&E (26).

### Analysis of the Gut Microbiota in Feces

Total DNA in feces was extracted using the FastDNA Spin Kit (MP Biomedicals, Santa Ana, CA, USA). The V3-V4 region of the 16S rRNA gene was amplified using 341F/806R primers. The library was built and sequenced on an Illumina MiSeq PE300 platform.

### Determination of SCFA Levels

Fecal samples (50mg) were dispersed in 50μL of saturated NaCl solution, acidified with 5% (v/v) H_2_SO_4_, and SCFAs were extracted with 1mL of diethyl ether. SCFA levels were tested by GC-MS (27).

### Statistical Analysis

Statistical analyses were performed using Prism version 7 (GraphPad, San Diego, CA, USA). Significant differences were evaluated using a one-way analysis of variance. Microbiota-related analyses, including alpha diversity and biodiversity richness, were assessed with the QIIME (version 1.17) and R (version 3.5.0) software.

## Results

### B[a]P-Binding Abilities

**Figure 1:**
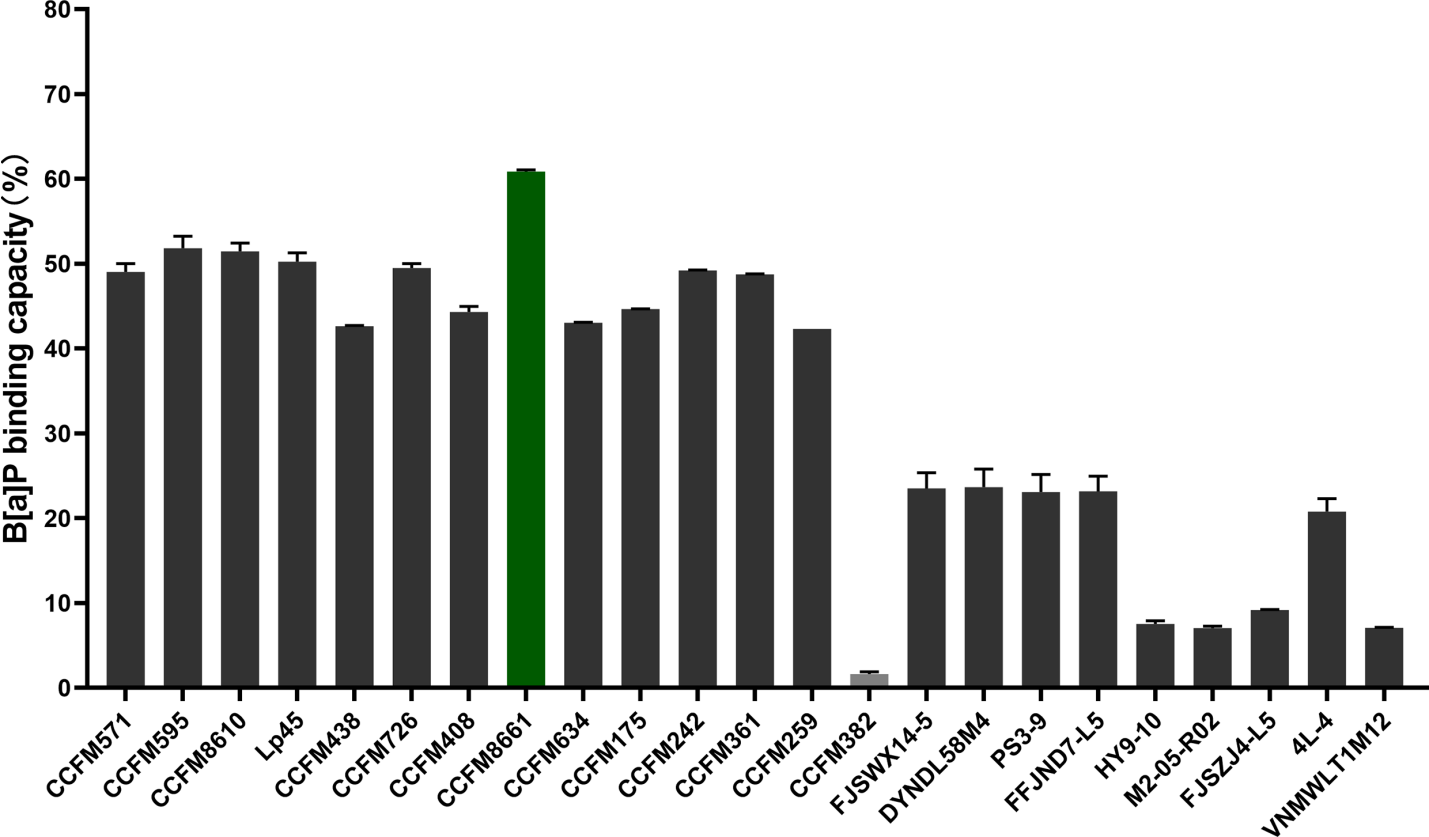
B[α]P-binding capacity of *L. plantarum* strains.

The B[a]P-binding abilities of the 23 *L. plantarum* strains are presented in **Figure 1**. The B[a]P-binding abilities were significantly different among the different *L. plantarum* strains. *L. plantarum* CCFM8661 had the highest B[a]P removal capacity, with a removal rate of 60.9%; thus, this strain was selected as the target strain for subsequent animal experiments. CCFM382 had the lowest removal rate of only 1.6%; thus, it was selected as the negative reference strain. The protection of these two *L. plantarum* strains against B[α]P-induced toxicity in the gut and the brain were compared.

### Levels of 3-OH B[α]P in Feces

**Figure 2:**
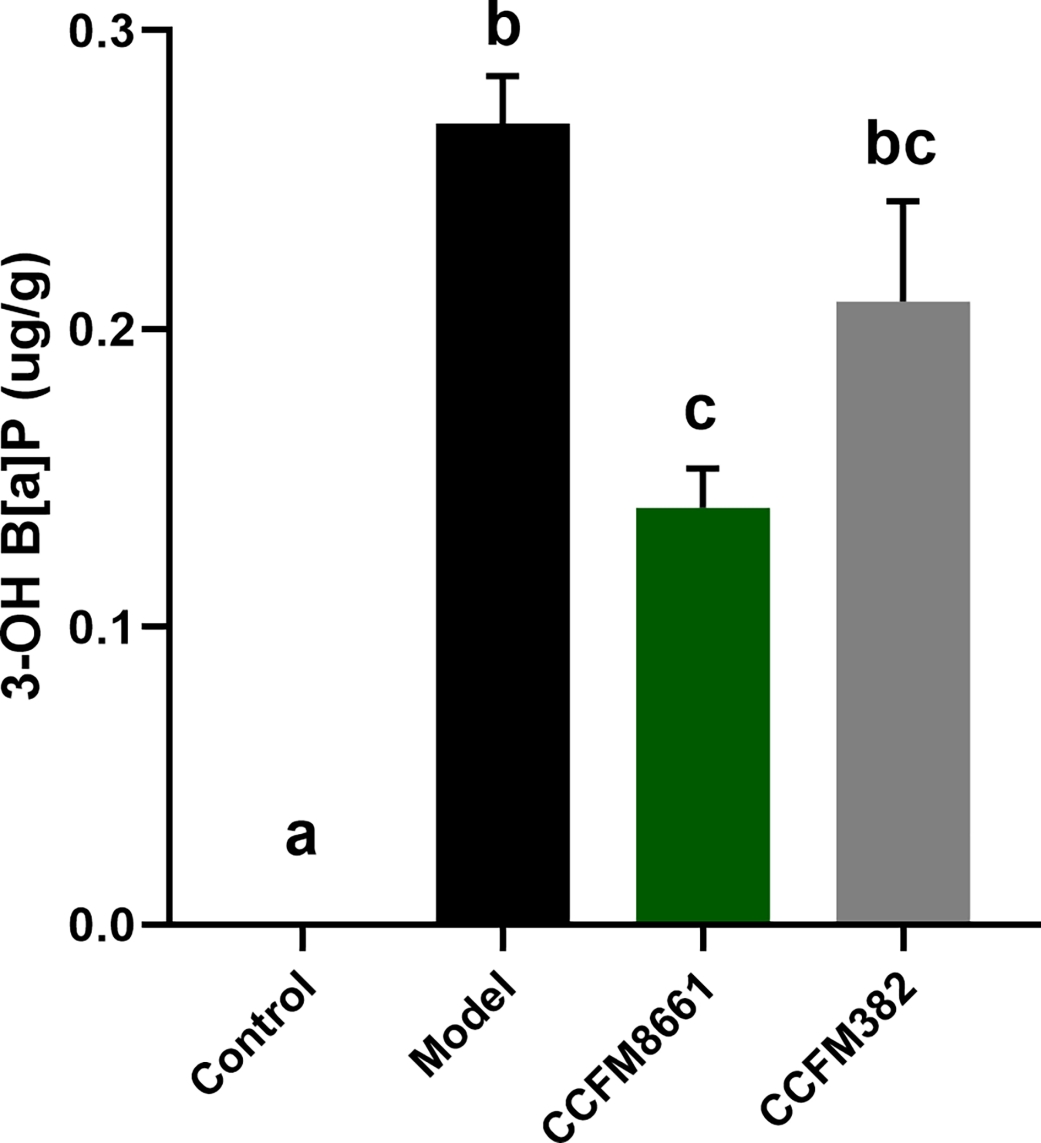
Effect of *L. plantarum* CCFM8661 on 3-OH B[α]P levels in feces. The different letters indicate significant difference between groups (*P < 0.05*).

The levels of 3-OH B[α]P in the feces of mice significantly increased after B[a]P treatment, which was up to 0.27 μg/g (**Figure 2**; *P*<0.05). The effects were significantly reversed by oral administration of *L. plantarum* CCFM8661 (*P*<0.05), but not *L. plantarum* CCFM382 (*P*>0.05). The 3-OH B[α]P level in the CCFM8661 and CCFM382 groups were 0.14μg/g, and 0.21μg/g, respectively.

### Open-Field Test

**Figure 3:**
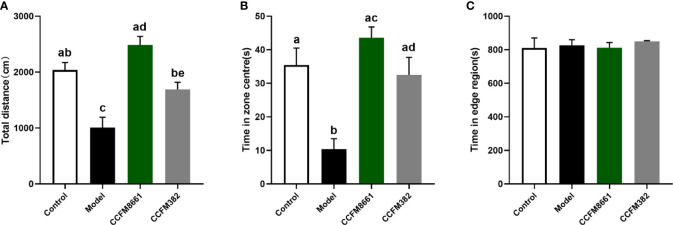
Effects of *L. plantarum* CCFM8661 on B[α]P-induced behavioral changes. **(A)** The total distance, **(B)** The time spend in zone centres, **(C)** The time spend in edge region. The different letters indicate significant difference between groups (*P < 0.05*).

The open-field test was used to evaluate the spatial cognitive ability of the experimental animals. The distance moved was used to represent autonomous activity ability, and the time spent in the central area was used to reflect their spatial cognition ability in a new environment. Mice with poor cognitive ability would quickly leave the central area and move along the periphery, thereby spending less exploration time in the central area. As shown in **Figure 3**, B[α]P-treated mice traveled a shorter total distance and spent less time in the center than mice in the control group (*P*<0.05). However, mice in the CCFM8661 and CCFM382 group traveled a longer distance and spent more time in the central zone than those in the model group (*P*<0.05). Importantly, *L. plantarum* CCFM8661 had a more significant increase on these two parameters than CCFM382. These results suggested that anxiety-like behavior caused by B[α]P can be better reversed by CCFM8661 supplementation.

### Oxidative Stress- and Tumor-Related Parameters in the Brain

**Figure 4:**
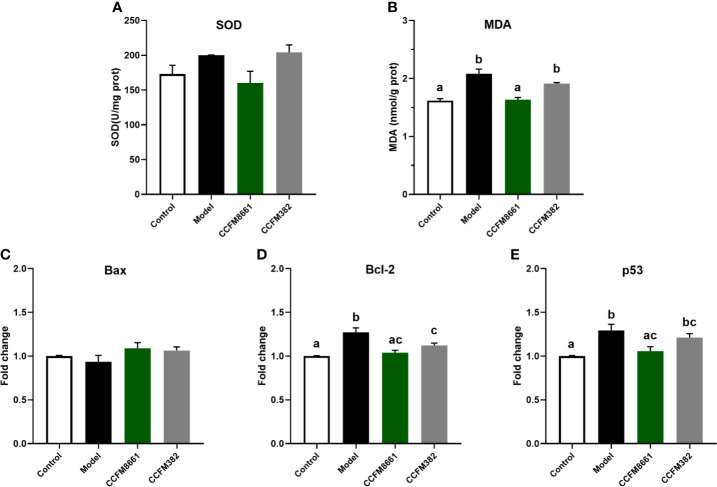
Effect of *L. plantarum* CCFM8661 on oxidative stress- and tumor-related parameters in the brain. **(A)** SOD activity, **(B)** MDA level, **(C–E)** mRNA expression levels of apoptosis-related genes in brain. The different letters indicate significant difference between groups (*P < 0.05*).

Malondialdehyde (MDA) levels were dramatically higher in the model group than those in the control group (**Figure 4B**, *P*<0.05). Of the two *L. plantarum* intervention groups, only *L. plantarum* CCFM8661 sharply decreased the MDA levels (*P*<0.05). Superoxide dismutase activity was not dramatically different between the groups (**Figure 4A**).

The expression levels of *Bcl-2* and *p53* in the brain increased dramatically in the model group, but *L. plantarum* CCFM8661 dramatically reduced the expression levels of these genes (**Figures 4D**, **E**; *P*<0.05). Although the decrease expression of *p53* in CCFM382 group was also observed, the difference was not significant (*P*>0.05). In addition, the *Bax* expression level in the brain tissue of the control, model, and two *L. plantarum*-intervention groups were no significant differences (**Figure 4C**).

### The mRNA Expression of TJ Proteins and *CYP1A1* in the Colon

**Figure 5:**
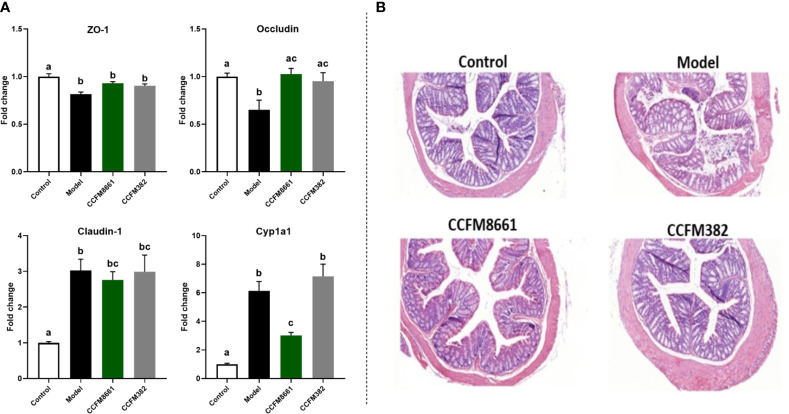
Effect of *L. plantarum* CCFM8661 on the parameters related to the colon. **(A)** The mRNA expression of TJ proteins and CYP1A1. **(B)** Histopathological changes. The different letters indicate significant difference between groups (*P < 0.05*).

The mRNA expression levels of *Zo-1* and occludin in the colon were dramatically lower in B[α]P-exposed mice than in control mice (**Figure 5A**; *P*<0.05). Oral administration of CCFM8661 and CCFM382 significantly increased occludin expression (*P*<0.05). The mRNA expression levels of claudin-1 were increased in the model group (*P*<0.05) but were not significantly affected by *L. plantarum* intervention (*P*>0.05).

Levels of the B[a]P-metabolizing enzyme CYP1A1, a member of the P450 enzyme family, were increased about six-fold after B[a]P treatment (**Figure 5A**; *P*<0.05), and *L. plantarum* CCFM8661, rather than *L. plantarum* CCFM382, significantly reduced its expression (*P*<0.05), thus alleviating the damage to the colon caused by B[a]P.

### Histopathological Changes in the Colon

Colonic histopathology was normal in mice in the control group (**Figure 5B**). However, B[α]P treatment led to serious injury to the colon, including crypt destruction, inflammatory cell infiltration, and severe ulceration. Colonic ulcers were reversed after *L. plantarum* CCFM382 administration, but there was still moderate inflammatory cell infiltration. Treatment with *L. plantarum* CCFM8661 significantly alleviated the colonic histopathological lesions to almost normal levels. Therefore, oral administration of *L. plantarum* CCFM8661 had a better alleviative effects on colonic damage induced by B[α]P.

### Gut Microbiota Diversity

**Figure 6:**
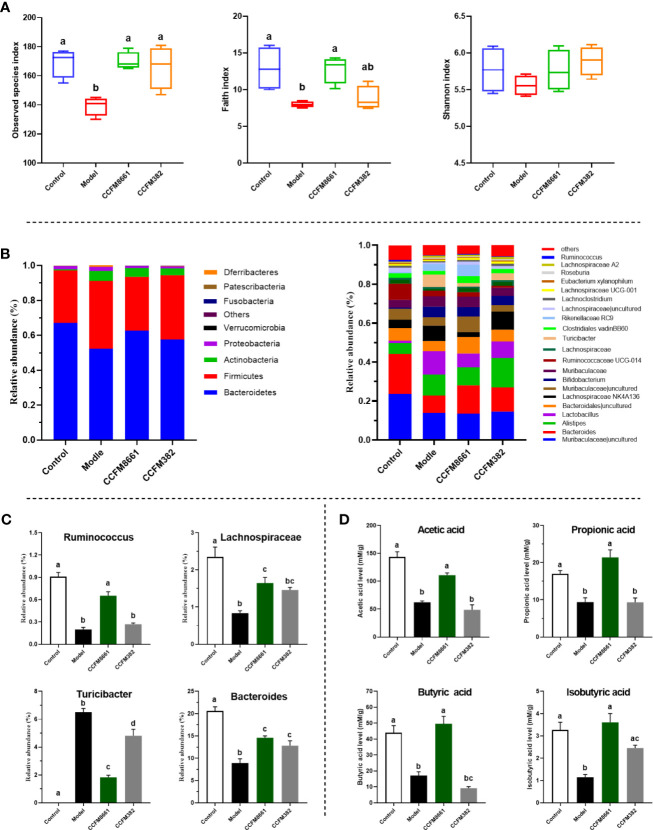
Effect of *L. plantarum* CCFM8661 on the diversity and composition of the gut microbiota and SCFA levels. **(A)** α-diversity, **(B)** relative abundance of constituents of the gut microbiota at the phylum and genus levels, **(C)** relative abundance of significant bacterial communities, **(D)** SCFA levels. The different letters indicate significant difference between groups (*P < 0.05*).

To determine whether the protective effects of *L. plantarum* CCFM8661 on B[α]P-induced colonic damage involved changes in the gut microbiota, the α diversity and composition of gut microbiota were measured. The number of observed species and the Shannon index were used to represent the gut microbiota richness and diversity, respectively. The Faith_pd index represents phylogenetic diversity, which is a qualitative measure of community richness. As shown in **Figure 6A**, the observed species and Faith_pd indices decreased significantly in the model group, indicating that the richness and diversity of the gut microbiota were dramatically reduced (*P*<0.05). Oral supplementation of *L. plantarum* CCFM8661 significantly increased these two indexes, but *L. plantarum* CCFM382 supplementation only elevated the observed species index (*P*<0.05). The results showed that *L. plantarum* CCFM8661 had a stronger regulating ability than CCFM382 on gut microbiota diversity.

### Composition of the Gut Microbiota

The α diversity results confirmed that B[α]P and *L. plantarum* CCFM8661 could, indeed, change the composition of the intestinal microbiota. Thus, changes in the gut microbiota composition were further explored (**Figure 6**). At the phylum level, Bacteroidetes, Firmicutes, Actinobacteria, and Proteobacteria accounted for more than 95% of the gut microbiota (**Figure 6B**). In the control group, these four predominant bacterial phyla accounted for 67.1%, 30.1%, 0.7%, and 1.8% of the gut microbiota, respectively. The abundance of Bacteroidetes, Firmicutes, and Actinobacteria in the model group was 52.2%, 38.9%, and 5.8%, respectively, whereas the abundance of Proteobacteria (2.3%) remained almost unchanged. After supplement with *L. plantarum* CCFM8661, the abundance of Bacteroidetes (62.7%), Firmicutes (30.8%) and Proteobacteria (1.1%) gradually recovered to the levels of the control group, while the abundance of Actinobacteria (5.0%) was similar to their abundance in the model group, without significant changes. The abundances of these four bacterial phyla in CCFM382 group accounted for 57.6%, 36.8%, 3.9% and 1.1% respectively. The results showed that CCFM382 also affected the gut microbiota composition, but the effects were not as strong as that of CCFM8661. At the genus level, *Muribaculaceae*, *Bacteroides*, *Alistipes*, and *Lachnospiraceae* NK4A136 accounted for 23.6%, 20.6%, 5.6%, and 4.4%, respectively, in the control group (**Figure 6B**). The abundance of *Muribaculaceae* and *Bacteroides* in the model group decreased to 13.8% and 8.9%, respectively, and the abundance of *Alistipes* and *Lachnospiraceae* NK4A136 increased to 10.8% and 7.9%, respectively, compared with those in the control group. In the CCFM8661 group, the abundance of *Bacteroides* increased to 14.6% and the abundance of *Lachnospiraceae* NK4A136 decreased to 2.5%. Moreover, after B[α]P treatment, the abundance of *Ruminococcus*, *Lachnospiraceae*, and *Bacteroides* decreased significantly and the abundance of *Turicibacter* increased (**Figure 6C**, *P*<0.05). *L. plantarum* CCFM8661 reversed the abundance of these four genera to their levels observed in the control group (*P*<0.05), while *L. plantarum* CCFM382 only reversed the abundances of *Turicibacter* and *Bacteroides*.

### SCFA Levels

Compared to the levels in the control group, the levels of SCFAs, including acetic acid, butyric acid, isobutyric acid and propionic acid, were dramatically reduced in mice treated with B[α]P (**Figure 6D**). *L. plantarum* CCFM8661 sharply increased the levels of these SCFAs *(P*<0.05*)* to levels almost the same as those in the control group. However, the administration of CCFM382 significantly increased only the levels of isobutyric acid among the SCFAs in B[α]P-treated mice (*P*<0.05). The results showed that *L. plantarum* CCFM8661 had a better effect than CCFM382 on increase of SCFA levels.

### Correlation and Heat Map Analyses

**Figure 7:**
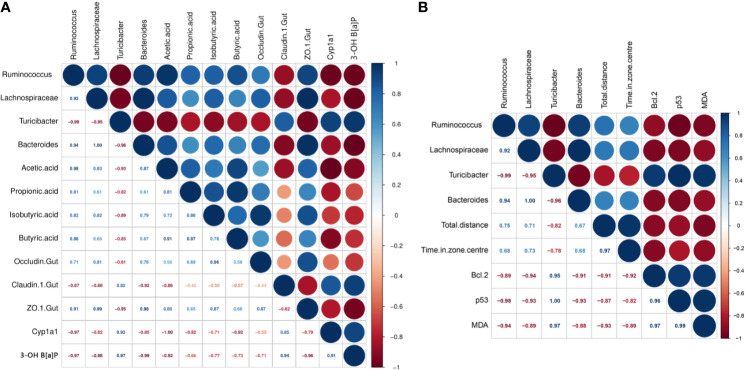
Correlations between parameters significantly affected by B[a]P and *L. plantarum* CCFM8661 treatment. **(A)** The correlation of intestinal microbiota with gut-related parameters, **(B)** The correlation of intestinal microbiota with brain-related parameters. Significant negative and positive correlations are represented by red and blue circles, respectively.

The parameters that were significantly affected by B[a]P and *L. plantarum* CCFM8661 were selected to assess their correlation using Pearson’s correlation coefficients (**Figure 7**). The *Zo-1* expression levels were positive correlated with the abundance of *Ruminococcus* (r=0.91), *Lachnospiraceae* (r=0.99), and *Bacteroides* (r=0.98), and negatively correlated with the *Turicibacter* abundance (r=-0.95), indicating that the gut microbiota was closely related to gut barrier function. The 3-OH B[a]P levels in feces had a strong positive correlation with *Turicibacter* abundance (r=0.97) but a negative correlation with the abundance of *Ruminococcus* (r=-0.97), *Lachnospiraceae* (r=-0.98), and *Bacteroides* (r=-0.99) and *Zo-1* expression levels (r=-0.96). These results confirmed that the gut microbiota and the gut barrier affected 3-OH B[a]P excretion in the feces, and *vice versa*. Moreover, *Bcl-2* expression levels showed a strong positive correlation with the abundance of *Turicibacter* (r=0.95) but a negative correlation with the abundance of *Lachnospiraceae* (r=-0.94) and *Bacteroides* (r=-0.91). *p53* levels were positively correlated with *Turicibacter* abundance (r=1.00) and negatively correlated with the abundance *Ruminococcus* (r=-0.98), *Lachnospiraceae* (r=-0.93) and *Bacteroides* (r=-0.93). MDA levels were positively correlated with *Turicibacter* abundance (r=0.97) and negatively correlated with the *Ruminococcus* abundance (r=-0.94), indicating that the gut microbiota was related to changes in tumor- and oxidative stress- related parameters in the brain.

**Figure 8:**
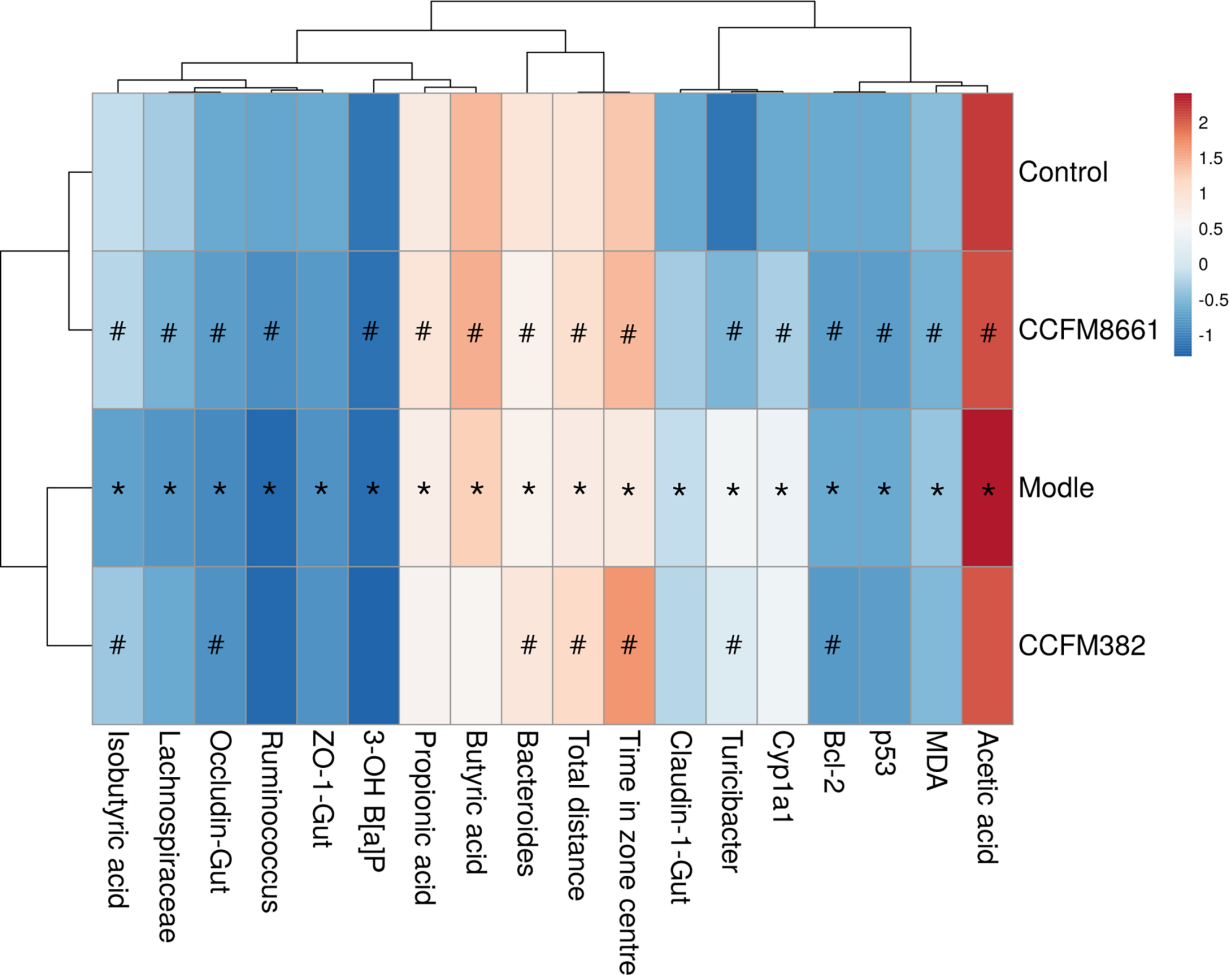
Clustered heat map of the parameters significantly affected by B[a]P and *L. plantarum* CCFM8661 treatment. Red indicates an increase in the corresponding group and blue indicates a decrease in the corresponding group. The asterisks indicate significant difference between control and model groups (*P < 0.05*). The pound signs indicate significant difference between model and *L. plantarum* intervention groups (*P < 0.05*).

Heatmap analysis was used to identify the similarities and differences among the four groups, as similar groups would cluster together in this analysis. As shown in **Figure 8**, the CCFM8661 treatment group was clustered with the control group, and the CCFM382 treatment group clustered with the model group, indicating that CCFM8661 had a strong protection against B[a]P-induced damage, which may almost recover to the levels of the control group, while CCFM8661 only had a little protective effects.

## Discussion

When performing *in vitro* screening of strains of the probiotic *L. plantarum* for the potential to alleviate B[α]P toxicity, the B[α]P-binding ability should be considered. The selected strain should have superb B[α]P-binding ability, allowing it to bind B[α]P before it is absorbed by the host intestine, thereby resulting in excretion of B[α]P *via* the feces. In this study, the B[α]P-binding capacities varied among the 23 *L. plantarum* strains tested, and CCFM8661 was found to have the highest B[α]P-binding ability. The adsorption of B[α]P and other carcinogens by *Lactiplantibacillus* strains has previously been reported, and the mechanisms may involve the adsorption of polysaccharides and proteins on the cell surface (18, 19, 28). The main metabolic product of B[α]P *in vivo* is 3-OH B[α]P. The levels of 3-OH B[α]P in the feces of mice treated with B[α]P were decreased after *L. plantarum* CCFM8661 supplementation, indicating that this strain may bind to B[α]P *in vivo* and, thereby, reduce its metabolism. It has been reported that probiotic *Lactiplantibacillus* spp. can bind to heavy metals, such as lead (29) and cadmium (30), and mycotoxins, such as aflatoxin (31), *in vivo*, thereby alleviating their toxic effects.

B[a]P treatment disturbed the balance of the gut microbiota. At the phylum level, B[a]P elevated the abundance of Proteobacteria and Firmicutes and reduced the abundance of Bacteroidetes. It has been reported that an increase in Proteobacteria and a decrease in Bacteroidetes are associated with polyaromatic hydrocarbons (32, 33). At the genus level, the abundance of the beneficial bacteria Clostridiales, *Lachnospiraceae*, *Ruminococcus*, and *Bacteroides* decreased, and the abundance of the pro-inflammatory bacteria *Turicibacter* spp. increased significantly. However, *L. plantarum* CCFM8661 supplementation reversed these effects. A previous study also reported that *L. salivarius* Ls-33 can change the relative abundance of *Clostridium* spp. in the feces of obese juveniles (34). Some Clostridiales members produce butyrate, which may have an anti-inflammatory effect (13), and its levels usually decrease in an unhealthy state (35). Members of the family *Lachnospiraceae* are the most important butyrate-producing microorganisms in the intestine, as they regulate host energy metabolism and mucosal integrity and create a proinflammatory environment (36), while *Ruminococcus* has the ability to regulate mucin expression and mucosal glycosylation in the colonic mucosa (37). The significant changes observed in the abundance of these bacteria indicated that B[a]P treatment can disrupt the gut microbiota composition, and *L. plantarum* CCFM8661 intervention can improve the homeostasis of the intestinal microbiota.

SCFAs are the metabolites of the gut microbiota, which can directly provide energy for intestinal epithelial cells and affect intestinal immunity and barrier function (38, 39). In this study, B[a]P exposure led to a decrease in the expression levels of the TJ-related genes *Zo-1* and occludin; this decrease may be attributed to the decrease in butyric and isobutyric acid production caused by B[a]P. *L. plantarum* CCFM8661 supplementation significantly increased the expression levels of occludin to almost normal levels. Previous studies have also reported that *Lactiplantibacillus* intervention can induce colonic TJ-related protein expression (40). In summary, B[a]P can significantly reduce the diversity of the intestinal microbiota, thus increasing the permeability of the intestinal barrier. Notably, *L. plantarum* CCFM8661 protected the integrity of the colonic mucosa and alleviated the pathological damage to the colon, which may have been related to changes in the intestinal microbiota. In addition, *L. plantarum* CCFM8661 significantly reduced the level of CYP1A1 induced by B[a]P and, thus, exerted a protective effect on the colon. Pithva et al. demonstrated similar results, showing that *L. rhamnosus* Vc alleviates colon injury caused by the carcinogen N-methyl-N ‘-nitroguanidine (41).

Mood and behavior are also closely related to changes in the intestine microbiota (42). B[a]P treatment resulted in disturbances of the gut microbiota, resulting in brain injuries and behavioral abnormalities. However, *L. plantarum* CCFM8661 reversed these effects. Bcl-2 has anti-apoptotic roles, and its overexpression can reduce the production of oxygen free radicals and lipid peroxides and inhibit changes in mitochondrial permeability, thus inhibiting apoptosis. Exposure to B[a]P and the pesticide triazophos has been shown to significantly increase *Bcl-2* expression levels in the brain (43). The tumor suppressor gene *p53* is one of the most frequently mutated genes and is highly correlated with human cancers. Abnormal expression of this gene can be found in more than 50% of all malignant tumors. When DNA damage is minor, the *p53* gene helps the cell to repair itself; however, if the damage is severe or the DNA repair mechanism fails, *p53* induces apoptosis. Intraperitoneal injection of B[α]P has been shown to increase the expression levels of *p53* in the cerebral cortex and hippocampus (44). *Lactiplantibacillus* administration has been shown to protect the brain from B[α]P-induced injury by reducing the expression levels of *Bcl-2* and *p53.* In addition, an increase in intestinal permeability induces systemic inflammation, causing an inflammatory response in the brain. The levels of MDA, an oxidative stress factor, reflect lipid peroxidation levels, which indicate the extent of injury to cells (45). A previous study also showed that B[α]P exposure increases MDA levels in the brain (46), indicating ROS generation and brain injury (47). Reportedly, *L. plantarum* strains have antioxidant abilities and induce a decrease in MDA levels (48).

In present study, the protection of two *L. plantarum* strains, with the highest and lowest B[a]P-binding ability respectively, against B[a]P-induced toxicity were compared. In terms of decreasing 3-OH B[a]P level in feces, improving colonic histopathology, oxidative stress- and tumor-related parameters in the brain and behavioral performance, regulating diversity and composition of gut microbiota and increasing SCFAs level, *L. plantarum* CCFM8661 performed better than *L. plantarum* CCFM382. The results of Heatmap analysis also proved this viewpoint. The underlying reasons for these different effects may main involve their significant difference in B[a]P-binding ability. However, in the aspect of colonic TJs expression and gut microbiota diversity, *L. plantarum* CCFM382 and *L. plantarum* CCFM8661 had the similar performance, which may attribute to the antioxidative ability or other probiotic properties of *L. plantarum* CCFM382.

## Conclusion

**Figure 9:**
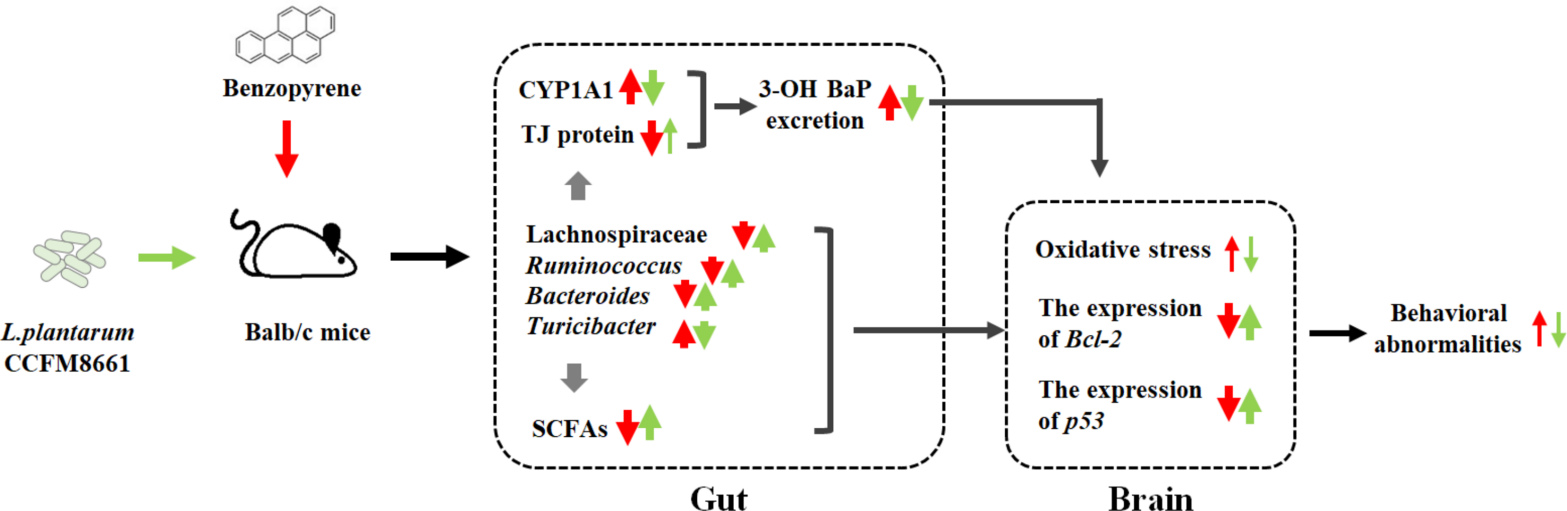
The possible protective mechanisms of *L. plantarum* CCFM8661 against B[a]P toxicity in the gut and brain. The red and green arrows represent the B[a]P- and CCFM8661-induced parameter changes, respectively. The thin arrows represent that only part of the parameter is affected.

Oral administration of *L. plantarum* CCFM8661 effectively alleviated colonic histopathological changes, reduced oxidative stress and tumor-related parameters in the brain, and improved behavioral performance. The underlying mechanism for these effects may involve the B[a]P-binding ability of CCFM8661 or the regulation of the gut microbiota by CCFM8661, including an increase in the gut microbiota diversity and the abundance of *Ruminococcus* and Lachnospiraceae and a decrease in the abundance of the pro-inflammatory *Turicibacter* spp., thereby increasing the SCFA levels and improving the integrity of the gut barrier (**Figure 9**). Therefore, oral administration of *L. plantarum* CCFM8661 is an effective, easy, and safe intervention against B[a]P-induced toxicity.

## Data Availability Statement

The data presented in the study are deposited in the NCBI repository, accession number from SRR15244744 to SRR15244788.

## Ethics Statement

All procedures and protocols of mice experiments were performed according to the guidelines of the Animal Care and Use Committee and the Ethics Committee of Jiangnan University (JN.No20190915b0481210).

## Author Contributions

LY: Methodology, Software, Formal analysis, Visualization, Funding acquisition, Writing—original draft. LZ: Investigation, Methodology, Software. HD: Writing—original draft, Methodology, Formal analysis. RZ: Investigation, Formal analysis. YX: Software, Software. MG: Investigation, Methodology. JZ: Supervision, Validation. HZ: Supervision, Validation. WC: Project administration, Funding acquisition. FT: Conceptualization, Writing—review and editing, Funding acquisition. All authors contributed to the article and approved the submitted version.

## Funding

This work was supported by National Natural Science Foundation of China Key Program (31772090, 32001665, 31820103010, U1903205), Natural Science Foundation of Jiangsu Province (BK20180603), the Key Scientific and Technological Research Projects (2018AB010), BBSRC Newton Fund Joint Centre Award, the National first-class discipline program of Food Science and Technology (JUFSTR20180102).

## Conflict of Interest

The authors declare that the research was conducted in the absence of any commercial or financial relationships that could be construed as a potential conflict of interest.

## Publisher’s Note

All claims expressed in this article are solely those of the authors and do not necessarily represent those of their affiliated organizations, or those of the publisher, the editors and the reviewers. Any product that may be evaluated in this article, or claim that may be made by its manufacturer, is not guaranteed or endorsed by the publisher.
